# Protodioscin protects porcine oocytes against H_2_O_2_-induced oxidative stress during *in vitro* maturation

**DOI:** 10.5713/ab.22.0282

**Published:** 2022-11-14

**Authors:** So-Hee Kim, Seung-Eun Lee, Jae-Wook Yoon, Hyo-Jin Park, Seung-Hwan Oh, Do-Geon Lee, Da-Bin Pyeon, Eun-Young Kim, Se-Pill Park

**Affiliations:** 1 Stem Cell Research Center, Jeju National University, Jeju 63243, Korea; 2 Faculty of Biotechnology, College of Applied Life Sciences, Jeju National University, Jeju 63243, Korea; 3 Mirae Cell Bio, Seoul, 04795, Korea; 4 Department of Bio Medical Informatic, College of Applied Life Sciences, Jeju National University, Jeju 63243, Korea

**Keywords:** Antioxidant, *In vitro* Maturation, Oxidative Stress, Protodioscin, Reactive Oxygen Species (ROS)

## Abstract

**Objective:**

The present study investigated whether protodioscin (PD), a steroidal saponin mainly found in rhizome of Dioscorea species, alleviates oxidative stress-induced damage of porcine oocytes during *in vitro* maturation.

**Methods:**

Oocytes were treated with different concentrations of PD (0, 1, 10, 100, and 200 μM) in the presence of 200 μM H_2_O_2_ during *in vitro* maturation. Following maturation, spindle morphology and mitogen-activated protein kinase activity was assessed along with reactive oxygen species level, GSH activity, and mRNA expression of endogenous antioxidant genes at the MII stage. On the day 7 after parthenogenetic activation, blastocyst formation rate was calculated and the quality of embryo and mRNA expression of development-related genes was evaluated.

**Results:**

Developmental competence was significantly poorer in the 0 μM PD-treated (control) group than in the non-treated (normal) and 10 μM PD-treated (10PD) groups. Although the reactive oxygen species level did not significantly differ between these three groups, the glutathione level and mRNA expression of antioxidant genes (superoxide dismutase 1 [*SOD1*], *SOD2*, nuclear factor erythroid 2-related factor 2 [*Nrf2*], and hemo oxygenase-1 [*HO-1*]) were significantly higher in the normal and 10PD groups than in the control group. In addition, the percentage of oocytes with defective spindle and abnormal chromosomal alignment was significantly lower and the ratio of phosphorylated p44/42 to total p44/42 was significantly higher in the normal and 10PD groups than in the control group. The total cell number per blastocyst was significantly higher in the 10PD group than in the control group. The percentage of apoptotic cells in blastocysts was highest in the control group; however, the difference was not significant. mRNA expression of development-related genes (POU domain, class 5, transcription factor 1 [*POU5F1*], caudal type homeobox 2 [CDX2], Nanog homeobox [NANOG]) was consistently increased by addition of PD.

**Conclusion:**

The PD effectively improves the developmental competence and quality of blastocysts by protecting porcine oocytes against oxidative stress.

## INTRODUCTION

*In vitro* embryo production is an important tool in agriculture, biomedical research, and assisted reproductive technology. Similar to the *in vivo* system, *in vitro* embryo production comprises three major consecutive steps: oocyte maturation, fertilization, and embryo culture. During oocyte development, unstable metabolites of oxygen known as reactive oxygen species (ROS) are generated as mitochondria produce energy via oxidative phosphorylation using oxygen or comes from their external environment [1]. An excessive level of ROS, i.e., an imbalance between endogenous antioxidant defense and ROS, leads to DNA fragmentation and apoptosis, and thereby decreases the developmental capacity of oocytes and blastocyst quality [2]. Thus, protection of oocytes against oxidative stress is important to improve the efficiency of *in vitro* embryo production [3]. Addition of antioxidants to media is one of the most fundamental and easiest strategies to improve embryo quality during *in vitro* culture [3].

Protodioscin (PD), a furostanol saponin obtained from the rhizome of Dioscorea species, has a wide array of biological activities such as anticancer, anti-inflammatory, and antioxidant effects [4]. PD reduces oxidative stress, as demonstrated by increases of superoxide dismutase (SOD) and glutathione (GSH) peroxidase activities, and decreases of ROS and malondialdehyde levels in neural cells [5]. In addition, inhibition of oxidative stress and apoptosis was observed together with increased expression of heat shock proteins in the presence of PD [5]. Although several studies reported that PD has beneficial effects on oxidative stress-induced damage and transcriptional regulation in neural cells [5,6], very little is known about the effect of PD in oocytes.

In the present study, we hypothesized that PD protects porcine oocytes against H2O2-induced oxidative stress during *in vitro* maturation (IVM) and investigated the quality of oocytes and embryos obtained by parthenogenetic activation (PA). Furthermore, we evaluated the ROS level, GSH activity, and mRNA expression of endogenous antioxidant and development-related genes. Cytoplasmic and nuclear maturation was also assessed to better understand the beneficial effects of PD. These findings may help to develop embryo production technology by facilitating further research of the mechanism via which PD inhibits oxidative stress in germ cells.

## MATERIALS AND METHODS

### Chemicals and reagents

All chemicals and reagents were purchased from Sigma (St. Louis, MO, USA) unless stated otherwise.

### *In vitro* maturation of porcine oocytes

Ovaries used in the experiment were obtained from pigs raised about 6 months and weigh approximately 120 kg. Prepubertal porcine ovaries were collected from a local slaughterhouse and transported to the laboratory in saline supplemented with 75 μg/mL penicillin G and 50 μg/mL streptomycin sulfate within 2 h at 30°C to 33°C. Cumulus-oocyte complexes (COCs) were aspirated from follicles with a diameter of 2 to 8 mm using an 18-gauge needle and a disposable 10 mL syringe. COCs were washed three times in tissue culture medium (TCM)-199–HEPES containing 0.1% (w/v) bovine serum albumin (BSA). Thereafter, COCs were matured in groups of 50 in 500 μL TCM-199 (Gibco, Grand Island, NY, USA) containing Earle’s salts, 0.57 mM cysteine, 10 ng/mL epidermal growth factor, 0.5 μg/mL follicle-stimulating hormone from porcine pituitary, 0.5 μg/mL luteinizing hormone from sheep pituitary, and 10% (v/v) porcine follicular fluid under mineral oil for 44 h at 38.8°C in 5% CO_2_ in air. Various concentrations (0 [control group], 1, 10, 100, and 200 μM) of PD were added together with 200 μM H_2_O_2_. For the normal group, neither PD nor H_2_O_2_ was added. Each experiment was independently repeated six times, with 50 to 60 oocytes per experiment. All data are presented as the means±standard error of the mean.

### Parthenogenetic activation and embryo culture

The aim of PA is to simulate the fertilization process by artificial stimulation to activate oocytes under non-sperm conditions [7]. Without sperm intervention, it is more efficient in researching physiological events which occurs in oocyte itself during its activation [7]. Moreover, it allows us to investigate the role of maternal genomes in controlling early embryo development separately from paternal genomes [8]. The PA and subsequent embryo culture were performed as previously described [9]. Following maturation, cumulus cells were removed by pipetting in the presence of 1 mg/mL hyaluronidase for 2 to 3 min. PA was induced by treating oocytes with porcine zygote medium-5 containing 0.4% (w/v) BSA (*in vitro* culture [IVC] medium) and 5 μM Ca^2+^ ionomycin for 5 min. After 3 h of culture in IVC medium containing 7.5 μg/mL cytochalasin B, embryos were washed three times with IVC medium and cultured for 7 days in the same medium at 38.8°C in a humidified atmosphere of 5% CO_2_ and 95% air. On day 5, half the medium was removed and replaced with PZM-5 containing 10% (v/v) fetal bovine serum. On day 7, blastocysts were washed in Dulbecco’s phosphate-buffered saline (DPBS), and either fixed in 3.7% (w/v) paraformaldehyde for 20 min and stored at 4°C, or lysed and snap-frozen in liquid nitrogen and stored at −80°C, depending on the experiment.

### Measurement of intracellular reactive oxygen species and glutathione levels

Dichlorohydrofluorescein diacetate (DCFHDA) and CellTracker Blue 4-chloromethyl-6,8-difluoro-7-hydroxy-coumarin (CMF_2_HC) were used to determine the intracellular levels of ROS and GSH, respectively, as previously described [10,11] with slight modifications. Briefly, cumulus cells were removed from COCs by pipetting in the presence of 0.1% (w/v) hyaluronidase. Denuded oocytes were incubated in DPBS containing 50 μM DCFHDA or 100 μM CMF_2_HC in the dark for 20 min at 38.8°C. Thereafter, oocytes were washed more than five times with DPBS containing 0.1% (w/v) BSA to completely remove excess dye and immediately analyzed by epifluorescence microscopy (Olympus, Tokyo, Japan). The ROS level was measured using excitation and emission wavelengths of 450 to 490 nm and 515 to 565 nm, respectively. The excitation and emission wavelengths of CMF_2_HC are 371 and 464 nm, respectively. Grayscale images were acquired with a digital camera (Nikon, Tokyo, Japan) attached to the microscope, and mean grayscale values were calculated using ImageJ software (NIH, Bethesda, MD, USA). Background fluorescence values were subtracted from the final values before statistical analysis. Each experiment was independently repeated 6 to 7 times, with 20 to 30 oocytes per experiment.

### Immunofluorescence

Meiotic spindles and nuclei of oocytes were visualized after maturation. Cumulus cells were removed from porcine COCs matured for 44 h and then oocytes were fixed overnight at 4°C in 4.0% (w/v) paraformaldehyde prepared in phosphate-buffered saline (PBS). Fixed oocytes were incubated for 30 min at 38.8°C with 0.5% (v/v) Triton X-100. After blocking for 1 h with 1% BSA (w/v) prepared in PBS (blocking solution I), oocytes were incubated overnight at 4°C with a mouse monoclonal anti-α-tubulin-FITC antibody (diluted 1:200 in blocking solution I). Nuclei were stained with Hoechst 33342 (1 μg/mL) for 30 min. Finally, oocytes were washed three times with PBS containing 0.1% (w/v) BSA, mounted on glass slides, and observed under an inverted Olympus IX-71 microscope. To further investigate the effect of PD on spindle organization, spindles without abnormalities were classified as normal, whereas those in which chromosomes failed to align at the metaphase plate were classified as abnormal [12]. Each experiment was independently repeated three times, and at least 20 oocytes were examined per group.

### Terminal deoxynucleotidyl transferase dUTP nick-end labeling and Hoechst staining

On day 7 after PA, blastocysts were fixed overnight at 4°C with 4.0% (w/v) paraformaldehyde prepared in PBS, washed three times with PBS containing 0.1% BSA, and then incubated with 0.1% Triton X-100 at 38.8°C for 30 min. Blastocysts were incubated with fluorescein-conjugated dUTP and terminal deoxynucleotidyl transferase (*In Situ* Cell Death Detection Kit; Roche, Manheim, Germany) in the dark for 1 h at 38.8°C. Thereafter, nuclei were stained with Hoechst 33342 (1 μg/mL) for 30 min, and stained blastocysts were washed with PBS containing 0.1% BSA. Washed blastocysts were mounted on glass slides and examined under an inverted Olympus IX-71 fluorescence microscope. The experiment was independently repeated 7 to 8 times, and at least 10 to 20 blastocysts were examined per group.

### mRNA extraction and complementary DNA synthesis

mRNA was isolated from more than three biological replicates, with 30 to 40 oocytes per replicate, using a Dynabeads mRNA Direct Kit (Invitrogen, Carlsbad, CA, USA) according to the manufacturer’s instructions. mRNA was collected in 10 μL elution buffer provided with the kit. Eluted RNA was reverse-transcribed into complementary DNA using an oligo (dT) 20 primer and SuperScript II reverse transcriptase (Invitrogen, USA) according to the manufacturer’s instructions.

### Real-time reverse transcription polymerase chain reaction

The protocol used was basically the same as that described previously [13]. Real-time reverse transcription polymerase chain reaction (RT-PCR) was performed using the primer sets listed in Table 2 and a StepOnePlus Real-time PCR System (Applied Biosystems, Warrington, UK) with a final reaction volume of 20 μL containing SYBR Green PCR Master Mix (Applied Biosystems, UK). The PCR conditions were as follows: 10 min at 95°C, followed by 39 cycles of 15 s at 95°C and 60 s at 54°C or 60°C. Samples were then cooled to 12°C. Relative gene expression levels were analyzed by the 2^−ΔΔCt^ method [14] after normalization against the expression level of the housekeeping gene *β-actin*. The experiment was independently repeated five times.

### Western blot analysis

The protocol was basically the same as that described previously [13]. In brief, oocytes (40 per sample) were solubilized in 20 μL of 1× sodium dodecyl sulfate (SDS) sample buffer (62.5 mM Tris-HCl, pH 6.8, containing 2% [w/v] SDS, 10% [v/v] glycerol, 50 μM dithiothreitol, and 0.01% [w/v] bromophenol blue or phenol red) and heated for 5 min at 95°C. Proteins were resolved on 5% to 12% Tris SDS-polyacrylamide gel electrophoresis gels for 1.5 h at 80 to 100 V. Samples were then transferred to Hybond-ECL nitrocellulose membranes (Amersham, Buckinghamshire, UK) at 300 mA for 2 h in transfer buffer (25 mM Tris, pH 8.5, containing 200 mM glycine and 20% [v/v] methanol). After blocking with 5% (w/v) nonfat milk prepared in PBS for 1 h, the membranes were incubated for at least 2 h with an rabbit anti-p44/42 mitogen-activated protein kinase (MAPK) or anti-phospho-p44/42 MAPK antibody both purchased from Cell Signaling Technology (diluted 1:500 in blocking solution [1× Tris-buffered saline, pH 7.5, containing 0.1% (v/v) Tween-20% and 5% (w/v) nonfat milk]). Thereafter, the membranes were washed three times in TBST (20 mM Tris-HCl, pH 7.5, containing 250 mM NaCl and 0.1% [v/v] Tween-20) and incubated for 1 h with mouse anti-rabbit IgG-horseradish peroxidase purchased from Santa Cruz Biotechnology (diluted 1:2,000 in blocking solution). After three washes with TBST, immunoreactive protein bands were visualized on X-ray films using the chemiluminescent reagent luminol (Invitrogen, USA) in a dark room. The experiment was independently repeated three times.

### Statistical analysis

The general linear model procedure within the Statistical Analysis System (SAS User’s Guide, 1985; Statistical Analysis System Inc., Cary, NC, USA) was used to analyze data from all experiments. The paired Tukey’s multiple range test was used to determine significant differences. p-values less than 0.05 were defined as statistically significant.

## RESULTS

### Protodioscin enhances *in vitro* development of porcine oocytes exposed to oxidative stress

To determine the optimal concentration of PD, porcine oocytes were matured for 44 h with 0, 1, 10, 100, and 200 μM PD (control, 1PD, 10PD, 100PD, and 200PD groups, respectively) in the presence of 200 μM H_2_O_2_. Oocytes in the normal group were matured in IVM medium without any supplements. Following PA, the percentage of cleaved oocytes on day 2 did not significantly differ between the groups (normal, 80.1%±2.5%; control, 78.6%±2.3%; 1PD, 74.8%±4.0%; 10PD, 78.4%± 4.0%; 100PD, 80.7%±3.6%; and 200PD, 81.9%±2.6%; Table 2). However, the percentage of oocytes that reached the blastocyst stage on day 7 was significantly higher in the normal and 10PD groups than in the control and 1PD groups, but did not significantly differ between these four groups and the 100PD and 200PD groups (normal, 38.9%±1.4%; control, 30.5%±3.3%; 1PD, 30.8%±3.4%; 10PD, 41.8%±2.9%; 100PD, 35.8 %±4.7%; and 200PD, 38.5%±3.8%; Table 2). Therefore, the normal, control, and 10PD groups were compared in subsequent experiments.

### Protodioscin protects porcine oocytes against oxidative stress

**Figure 1:**
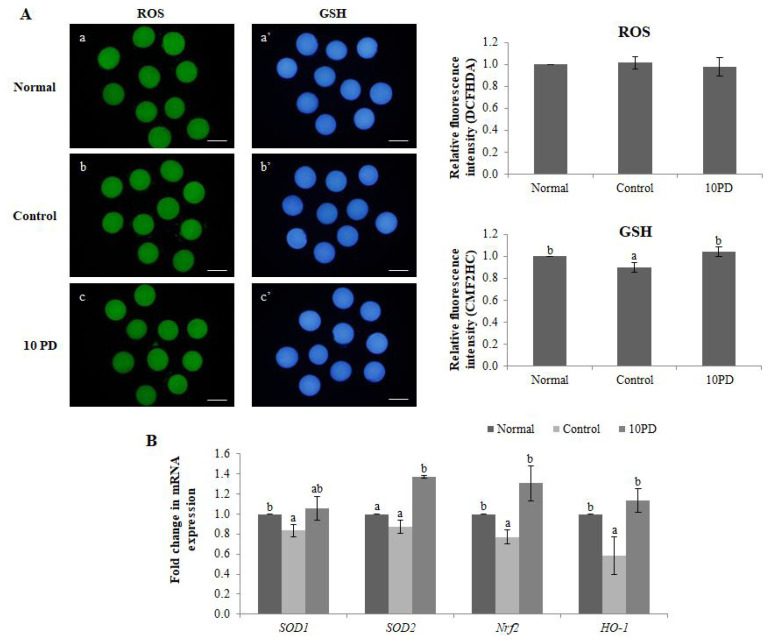
Antioxidant effect of PD on porcine oocytes *in vitro*. (A) Epifluorescence images of oocytes stained with DCFHDA (left) and CellTracker Blue CMF_2_HC (right), and the fluorescence intensities of intracellular ROS and GSH staining. a and a′: normal group; b and b′: control group; and c and c′: 10PD group. a, b, and c: ROS staining; a′, b′, and c′: GSH staining. (B) Relative expression of the antioxidant genes *SOD1*, *SOD2*, *Nrf2*, and *HO-1*. Data were derived from 3 to 8 independent replicates per group. Data are the means±SEM. PD, protodioscin; *SOD1*, superoxide dismutase1; *Nrf2*, nuclear factor erythroid 2-related factor 2; *HO-1*, hemo oxygenase-1. ^a,b^ p<0.05. Scale bar = 120 μm.

The effects of PD on the ROS and GSH levels were assessed by staining oocytes with DCFHDA and CMF_2_HC, respectively (Figure 1A). The ROS level did not significantly differ between the three groups. The GSH level was significantly higher (p<0.05) in the normal and 10PD groups than in the control group.

Expression of the antioxidant genes *SOD1*, *SOD2*, nuclear factor erythroid 2-related factor 2 (*Nrf2*), and hemo oxygenase-1 [*HO-1*] was analyzed by real-time RT-PCR (Figure 1B). mRNA expression of *SOD1* was significantly higher (p<0.05) in the normal group than in the control group, and was substantially higher in the 10PD group than in the control group; however, this difference was not significant. mRNA expression of *SOD2* was significantly higher (p<0.05) in the 10PD than in the normal and control groups, but did not significantly differ between the latter two groups. mRNA expression of *Nrf2* was significantly higher in the normal and 10PD groups than in the control group. The mRNA expression pattern of *HO-1* was similar to that of *Nrf2*.

### Protodioscin prevents aberrant spindle formation and abnormal chromosomal alignment in porcine oocytes exposed to oxidative stress

**Figure 2:**
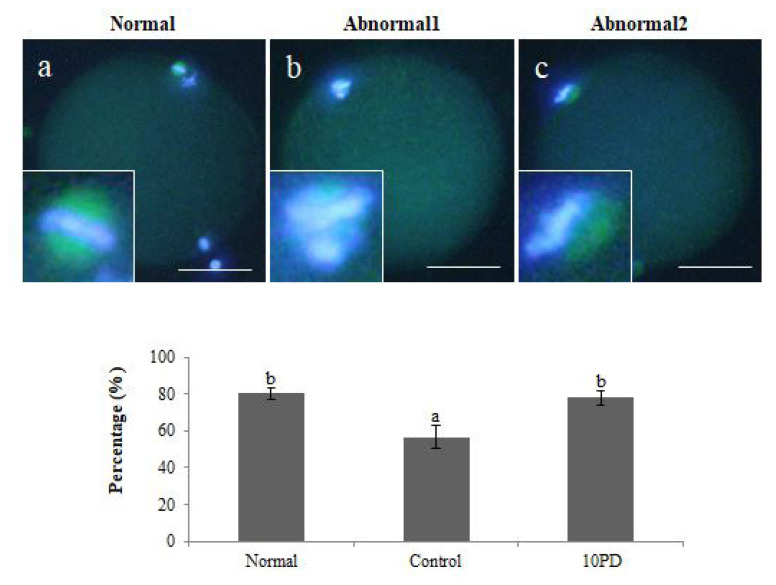
Effect of protodioscin (PD) on meiotic spindle morphology in porcine oocytes *in vitro*. Normal and abnormal chromosomal alignment and meiotic spindle formation in oocytes and percentage of oocytes in which the morphologies of chromosomes and the meiotic spindle were normal. Data were derived from 3–4 independent replicates per group. Data are the means±standard error of the mean. ^a,b^ p<0.05. Scale bar = 50 μm.

The percentage of oocytes with a normal meiotic spindle and normal chromosomal alignment was significantly higher in the normal (p<0.01) and 10PD (p<0.05) groups than in the control group, and was similar in the normal and 10PD groups (normal, 80.5%±3.1%; control, 56.8%±6.4%; and 10PD, 78.0%±4.1%; Figure 2).

### Protodioscin increases expression of a cytoplasmic maturation marker in porcine oocytes exposed to oxidative stress

**Figure 3:**
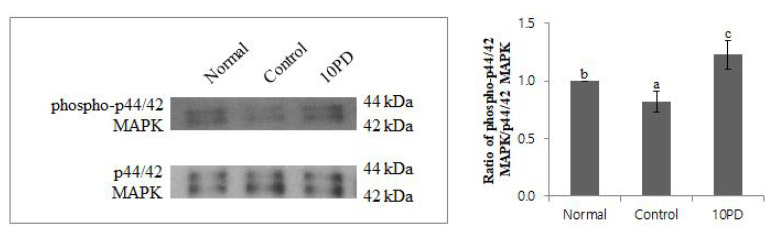
Effect of protodioscin (PD) treatment of porcine oocytes *in vitro* on mitogen-activated protein kinase activity. Data were normalized against the levels in the control group and were derived from 6 to 7 independent replicates per group. Data are the means±standard error of the mean. ^a–c^ p<0.05.

Several studies have suggested that MAPK phosphorylation is an important marker to evaluate cytoplasmic maturation. Therefore, we investigated whether PD improves porcine oocyte maturation via the MAPK signal transduction pathway. Lysates from the normal, control, and 10PD groups were immunoblotted with an anti-phosphorylated MAPK antibody and subsequently re-probed with an anti-MAPK antibody to normalize the densitometric results. MAPK migrates as a doublet at 44 and 42 kDa, representing p44/42 MAPK (ERK1/2). The ratio of phosphorylated MAPK (phospho-p44/42 MAPK), which is the active form, to total MAPK was significantly lower (p<0.05) in the control group than in the normal and 10PD groups and was significantly higher (p<0.05) in the 10PD group than in the normal group (normal, 1.0±0.0; control, 0.8±0.1; and 10PD, 1.2±0.1; Figure 3).

### Protodioscin improves the quality of blastocysts derived from porcine oocytes exposed to oxidative stress *in vitro*

**Figure 4:**
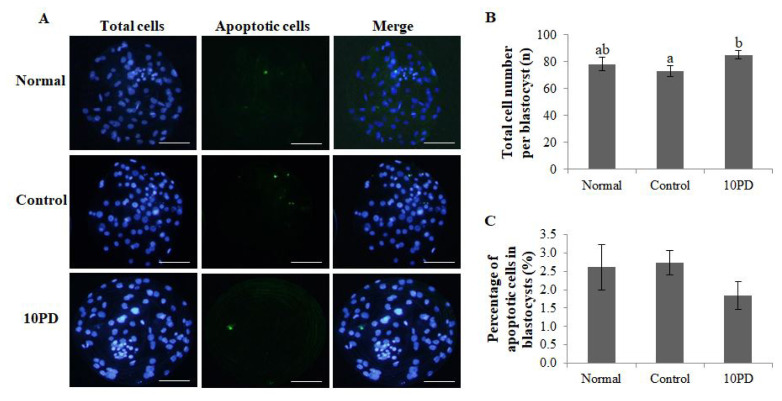
Effect of protodioscin (PD) treatment of porcine oocytes *in vitro* on subsequent embryo quality after PA. (A) Blastocyst staining. (B) Total cell number per blastocyst. (C) Percentage of apoptotic cells in blastocysts. Data were derived from 7 to 8 independent replicates per group. Data are the means±standard error of the mean. ^a,b^ p<0.05. Scale bar = 50 μm.

To investigate whether PD treatment during IVM of porcine oocytes influences subsequent embryo development and quality, the total cell number and genomic DNA fragmentation in blastocysts were assessed (Figure 4A). The total cell number per blastocyst was significantly higher (p<0.05) in the 10PD group than in the control group, and was slightly lower in the control group than in the normal group; however, this difference was not significant (normal, 78.0±5.0; control, 73.2±4.1; and 10PD, 85.0±2.9; Figure 4B). The percentage of apoptotic cells in blastocysts determined by assessment of genomic DNA fragmentation was higher in the control group than in the normal and 10PD groups; however, this difference was not significant (normal, 2.6%±0.6%; control, 2.7%±0.3%; and 10PD, 1.8%±0.4%; Figure 4C).

### Protodioscin alters expression of development-related genes in porcine oocytes exposed to oxidative stress

**Figure 5:**
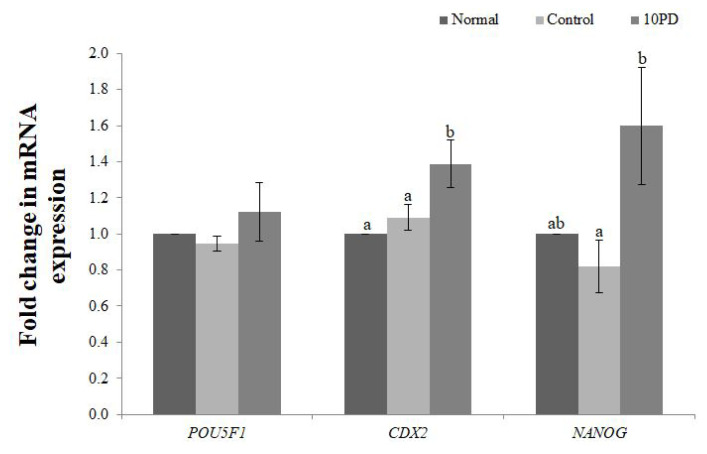
Effect of protodioscin (PD) treatment of porcine oocytes *in vitro* on expression of development-related genes. Data were derived from 3 to 4 independent replicates per group. Data are the means±standard error of the mean. ^a,b^ p<0.05.

Expression of the development-related genes (POU domain, class 5, transcription factor 1 [*POU5F1*], caudal type homeobox 2 [*CDX2*], Nanog homeobox [*NANOG*]) at the blastocyst stage was analyzed by real-time RT-PCR (Figure 5). mRNA expression of *POU5F1* was slightly decreased by addition of H_2_O_2_ and increased by supplementation of PD, but did not significantly differ between the three groups. mRNA expression of *CDX2* was significantly higher (p<0.05) in the 10PD group than in the normal and control groups, and was similar in the latter two groups. mRNA expression of *NANOG* was slightly lower in the control group than in the normal group, and was significantly higher (p<0.05) in the 10PD group than in the control group.

## DISCUSSION

Oxidative stress caused by ROS is an important cause of apoptosis, inhibition of oocyte maturation and early embryonic development [15]. Several studies have shown that PD elicits antioxidant effects and reduces apoptosis caused by oxidative stress in neural cells [5,6]. This study investigated the effects of PD on H_2_O_2_-induced oxidative stress in porcine oocytes. In the present study, we showed that addition of H_2_O_2_ during IVM significantly diminished the developmental capacity of porcine oocytes. However, supplementation of 10 μM PD significantly improved oocyte quality, which is impaired by H_2_O_2_ in a concentration-dependent manner, and consequently enhanced embryo development as reflected by the percentage of oocytes that reached the blastocyst stage (Table 1). Blastocyst formation is a critical indicator of the efficiency of embryo development and culture conditions [16]. Although the percentages of surviving oocytes at metaphase of the second meiotic division (MII) stage and cleaved oocytes did not significantly differ between the three groups, the percentage of oocytes that reached the blastocyst stage was significantly higher in the 10PD and normal groups than in the control group.

To find out whether changes in M II oocytes matured in an environment inducing oxidative stress affect subsequent embryo development, alterations in the ROS and GSH levels and spindle morphology of MII oocytes were investigated. Addition of PD to IVM medium containing H_2_O_2_ did not affect the ROS level in oocytes at the MII stage in comparison with the normal and control groups. However, the GSH level was significantly lower in the control group than in the normal group, and was slightly higher in the 10PD group than in the normal group. This suggests that the beneficial effect of PD on porcine oocytes is attributable to an increase in endogenous antioxidants rather than a decrease in the ROS content. Similarly, mRNA expression of antioxidant genes (*SOD1*, *SOD2*, *Nrf2*, and *HO-1*) was consistently lower in the control group than in the normal and 10PD groups. This demonstrates that H_2_O_2_ remarkably decreases transcription of antioxidant genes and PD alleviates the effects of H_2_O_2_ and upregulates relative mRNA expression of these genes.

During meiotic maturation, formation of the spindle is very important for alignment of chromosomes, which is directly related to separation of chromosomes and normal development of embryos in meiosis, and failure of this process results in genetic disorders and aneuploid embryos [17]. Several studies have shown that oxidative stress affects microtubule assembly in interphase cells, suggesting that ROS may affect spindle formation [18]. For example, a delay of spindle formation was observed in a study using HeLa cells exposed to oxidative stress. Moreover, the appearance of misaligned chromosomes and multipolar spindles in metaphase is substantially increased in the presence of H_2_O_2_ [19]. Likewise, in our study, the percentage of oocytes with normal spindle morphology was remarkably lower in the control group than in the normal and 10PD groups. Consistently, an aberrant configuration of chromosomes was observed more often in the control group than in the other groups. These results demonstrate that addition of H_2_O_2_ negatively affects spindle formation and leads to abnormal chromosomal alignment. However, PD attenuates the negative effects of H_2_O_2_ and promotes normal development of oocytes and embryos in meiosis.

The PD treatment also considerably increased the phos phorylated MAPK level. MAPK plays crucial roles in regulation of oocyte maturation along with MPF, which is a complex of cyclin B and Cdc2. MAPK plays a vital role in early embryo development processes, such as initiation of the first meiotic division in germinal vesicle stage, promotion of nuclear maturation, and oocyte maintenance at the MII stage [20]. Several studies also suggested that phosphorylation of MAPK is an important marker to evaluate cytoplasmic maturation along with cyclin B2 levels [21]. In our study, the increased level of phosphorylated MAPK suggests that PD enhances MAPK activity in H_2_O_2_-treated oocytes.

The percentage of blastocysts obtained by PA and the aver age total cell number per blastocyst were higher in the 10PD group than in the control group. The total cell number per blastocyst indicates the quality of blastocysts [22]. It is a standard criterion for evaluating the quality of embryos and indicates how well embryos are developed. By contrast, increased apoptosis is an important indicator of inadequate *in vitro* conditions for oocytes [23]. Apoptosis is a process of programmed cell death that occurs regularly to ensure a homeostatic balance between the rates of cell formation and cell death, and involves many genes. However, excessive apoptosis can induce degeneration of oocytes and death of early embryos, and also affect normal blastocyst formation [24]. In this study, the average percentage of apoptotic cells in blastocysts was lower in the 10PD group than in the normal and control groups; however, this difference was not significant.

To further understand the effect of PD on development of embryos, we assessed expression of development-related genes. *POU5F1* and *NANOG* play important roles in maintaining the pluripotency of embryonic stem cells and promoting cell proliferation [25]. Knockout of *POU5F1* and *NANOG* inhibits blastocyst development [26]. Similarly, *CDX2* is essential for viability and proliferation of blastocyst cells [27]. Expression of *POU5F1*, *CDX2*, and *NANOG* was consistently higher in the 10PD group than in the normal and control groups. This demonstrates that the improvement of early embryonic development by PD is closely correlated with upregulation of these genes.

Our data indicate that H _2_O_2_ negatively affects the development of oocytes and reduces the quality of embryos and blastocysts derived from these oocytes, while supplementation of PD improves the developmental rate and enhances the quality of oocytes, and increases expression of antioxidant and development-related genes. Our results also demonstrate that PD protects porcine oocytes against H_2_O_2_-induced oxidative stress by inducing production of several antioxidant enzymes, including *SOD1*, *SOD2*, *Nrf2*, and *HO-1*, and further promotes normal early embryo development by supporting meiosis, especially spindle formation, to occur at an appropriate time and in an appropriate manner. PD only subtly affected the ROS level and percentage of apoptotic cells in blastocysts in this study, but the impacts about which these differences brings may be substantial in terms of developing oocytes or embryos.

Considering the differences in experimental data between the three groups and previous findings, among the antioxidant genes, *Nrf2* probably play a pivotal role in the mechanism of action. *Nrf2* is well-known as a key transcription factor that induces the expression of antioxidant proteins [5]. Under normal conditions, *Nrf2* is inactivated as it is bound to the negative regulator called Kelch-like ECH2 associated protein 1 (*Keap1*) within the cytoplasm. However, under oxidative circumstances, It is released from *Keap1* and translocates into the nucleus, where it binds to antioxidant response elements [28]. As a result, activation of the genes which express antioxidant enzymes such as *SOD* and *HO-1*, and enzymes responsible for the GSH synthesis occurs [28]. *Nrf2* is also considered the downstream factor of *ERK1/2* (a.k.a P44/42 MAPK) activation [29]. Additionally, according to previous studies, it is suggested that *Nrf2* closely associated with the development-related genes such as *POU5F1* and *NANOG*, thus its inhibition impairs the regulation of self-renewal ability and pluripotency during cellular reprogramming [30]. In conclusion, present study suggests that PD ameliorates development potential of porcine oocytes under oxidative environment via *ERK/Nrf2/HO-1* signaling pathway. Further research is necessary to understand the details of the mechanisms by which PD affects development of porcine oocytes.

## Figures and Tables

**Table 1:** Primers used for real-time reverse transcription polymerase chain reaction

Gene	GenBank accession no.	Primer sequence	Annealing temperature (°C)	Product size (bp)
*β-actin*	AY550069.1	F: AGATCATGTTCGAGACCTTC R: GTCAGGATCTTCATGAGGTAGT	49	220
*SOD1*	GU944822.1	F: GTGTTAGTAACGGGAACCAT R: GGATTCAGGATTGAAGTGAG	54	120
*SOD2*	NM_214127.2	F: AGACCTGATTACCTGAAAGC R: CTTGATGTACTCGGTGTGAG	54	110
*Nrf2*	Gu991000.1	F: CTATGGAGACACACTGCTTG R: ACAGGCTGTGTTTTAGGACT	54	99
*HO-1*	NM001004027.1	F: ACCCAGGACACTAAGGACCA R: CGGTTGCATTCACAGGGTTG	54	227
*POU5F1*	NM_001113060	F: AGTGAGAGGCAACCTGGAGA R: TCGTTGCGAATAGTCACTGC	54	166
*CDX2*	AM778830	F: AGCCAAGTGAAAACCAGGAC R: TGCGGTTCTGAAACCAGATT	48	178
*NANOG*	DQ447201	F: TTCCTTCCTCCATGGATCTG R: ATCTGCTGGAGGCTGAGGTA	53	214

F, forward; R, reverse; *SOD1*, superoxide dismutase1; *Nrf2*, nuclear factor erythroid 2-related factor 2; *HO-1*, heme oxygenase-1; *POU5F1*, POU domain, class 5, transcription factor 1, *CDX2*, caudal type homeobox 2, *NANOG*; Nanog homeobox.

**Table 2:** Effect of PD treatment of porcine oocytes *in vitro* on subsequent embryo development

Treatment group	H_2_O_2_ concentration (μM)	PD concentration (μM)	No. of germinal vesicle oocytes	No. (%) of		

				Surviving oocytes^1)^	Cleaved oocytes on day 2^2)^	Blastocysts on day 7^3)^
Normal	0	0	300	282 (94.0±1.5)	226 (80.1±2.5)	88 (38.9±1.4)^b^
Control	200	0	300	280 (93.3±1.6)	220 (78.6±2.3)	67 (30.5±3.3)^a^
1PD	200	1	300	282 (94.0±1.3)	211 (74.8±4.0)	65 (30.8±3.4)^a^
10PD	200	10	300	287 (95.7±1.4)	225 (78.4±4.0)	94 (41.8±2.9)^b^
100PD	200	100	300	280 (93.3±1.3)	226 (80.7±3.6)	81 (35.8±4.7)^ab^
200PD	200	200	300	282 (94.0±1.5)	231 (81.9±2.6)	89 (38.5±3.8)^ab^

Values are means±standard error of the mean of independent experiments.

PD, protodioscin.

1) The percentage of oocytes that reached MII.

2) The percentage of oocytes that underwent cleavage.

3) The percentage of cleaved oocytes that reached the blastocyst stage on day 7.

a,b Values with different superscript letters are significantly different (p<0.05).
